# Shaping light in 3d space by counter-propagation

**DOI:** 10.1038/s41598-021-97313-4

**Published:** 2021-09-09

**Authors:** Ramon Droop, Eric Asché, Eileen Otte, Cornelia Denz

**Affiliations:** Institute of Applied Physics, University of Muenster, Corrensstr. 2/4, 48149 Münster, Germany

**Keywords:** Optical techniques, Other photonics

## Abstract

We extend the established transverse customization of light, in particular, amplitude, phase, and polarization modulation of the light field, and its analysis by the third, longitudinal spatial dimension, enabling the visualization of longitudinal structures in sub-wavelength (nm) range. To achieve this high-precision and three-dimensional beam shaping and detection, we propose an approach based on precise variation of indices in the superposition of higher-order Laguerre-Gaussian beams and cylindrical vector beams in a counter-propagation scheme. The superposition is analyzed experimentally by digital, holographic counter-propagation leading to stable, reversible and precise scanning of the light volume. Our findings show tailored amplitude, phase and polarization structures, adaptable in 3D space by mode indices, including sub-wavelength structural changes upon propagation, which will be of interest for advanced material machining and optical trapping.

## Introduction

Structured light is an ubiquitous phenomenon, that naturally occurs in Rayleigh-scattering of sunlight in the atmosphere^1,2^ or bundles of light rays focused into caustic structures by transparent media^3–5^, to name a few. The research on structured light aims for the spatial beam shaping in the different degrees of freedom, namely amplitude, phase and polarization. An established scalar beam class that exhibits a spatially variation in its amplitude and phase represents ring-shaped Laguerre-Gaussian beams that carry orbital angular momentum (OAM). These beams show a twisted, helical wave front due to their azimuthally varying transverse phase structure (phase vortex) with central phase singularity, and find applications in optical tweezers for orbiting or spinning particles^6–8^, or decreasing the fluorescence signal in the depletion step of STED microscopy^9^. Besides variation of scalar properties of light (amplitude and phase), more recent structuring of polarization leads to vectorial light fields that show spatially inhomogeneous polarization patterns, as cylindrical vector beams (CVBs)^10^, which may include polarization singularities^11–13^. In these fields the spin angular momentum (SAM) of light, transferable to optically trapped, birefringent particles^14^, can be shaped as it is connected to the polarization handedness. By tightly focusing vectorial fields, initially radially oriented electric field components flip into the longitudinal propagation direction, enabling adaptable optical forces in optical tweezers^15^, sub-wavelength spot sizes in a microscopy^16^, or advanced polarization landscapes including, for instance, polarization Möbius stripes^17,18^.

The majority of approaches for structuring light aims for precise patterning of the transverse dimensions, i.e. in two-dimensional (2d) space. This yields a good control of the characteristics of these light fields in a single plane, which is of particular interest for applications as optical tweezers, whose highest requirements are in the working plane of the microscope objective. However, light is naturally a spatially three-dimensional (3d) phenomenon and, thus, to fully understand it, it needs a 3d spatial analysis. Being able to specifically tailor and analyze such structures on demand is a challenging task, but if it succeeds, could advance material machining, particle trapping, and imaging applications from 2d to 3d space. Until now, shaping a single beam, different 3d structured light fields have been realized as non-diffracting^19–22^, accelerating^23^, or customized fields^24–27^ as for instance tractor beams, frozen waves or knotted intensity structures. Furthermore, driven by its implementation for optical trapping^28^, first approaches to shape light by counter-propagating beams have been presented. Taking advantage of fundamental or lower-order Bessel or Laguerre-Gaussian beams, counter-propagating optical trapping landscapes have been created, orbiting trapped particles or nanorods^29–31^. However, these light structures typically either lack transverse as well as longitudinal structural diversity, in particular, when considering the polarization of light as a degree of freedom, and/ or require complex realization procedures. Moreover, light structures shaped by counter-propagating light fields have typically only been verified indirectly by observing trapped objects, thus, lacking detection of its detailed 3D structure with high resolution up to now.

To address these shortcomings, we propose a straightforward, flexible approach based on the combination of the fundamental concept of counter-propagation with an innovative scanning method that enables high-resolution analysis of the respective 3d structured light field. For this purpose we superimpose two self-similar light fields in a counter-propagating configuration, which—in contrast to previous approaches—can be tailored individually in its amplitude, phase, and/ or polarization. They facilitate the access to a broad variety of extended light landscapes of sub-wavelength structures in a 3d volume. To experimentally demonstrate the 3d nature of structures at high spatial resolution, we implement a scanning approach based on digital, artificial counter-propagation of physically co-propagating beams by a spatial light modulator. The self-similarity of the implemented beams ensures the desired increase in longitudinal extent, since, apart from divergence, the superimposed beams stay structurally unchanged upon propagation. By adapting the mode indices of the employed self-similar fields, we simultaneously shape the relative transverse and longitudinal phase and/or polarization of counter-propagating beams. Thereby, we control and sculpt the transverse as well as the longitudinal degrees of freedom of the realized volumetric light field.

Our customization approach allows two different realizations to evince versatility and high experimental resolution. On the one hand, a Laguerre-Gaussian (LG) base is used, enabling a precise control of the transversely-resolved amplitude and phase in the generated light volume by mode indices. This approach allows also to include the until now neglected polarization degree of freedom, forming complex polarization patterns with dynamic propagation behavior. On the other hand, we use cylindrical vector beams (CVBs) as a base, leading to a beating between transverse phase and polarization structures along the propagation axis while the amplitude stays constant. This allows sculpting the transverse polarization structure of counter-propagating beams in a straightforward manner. Our flexible approach is of specific interest for next-generation counter-propagating optical trapping in 3d volumes, in particular, of polarization-sensitive particles.

## Results

### Concept

**Figure 1:**
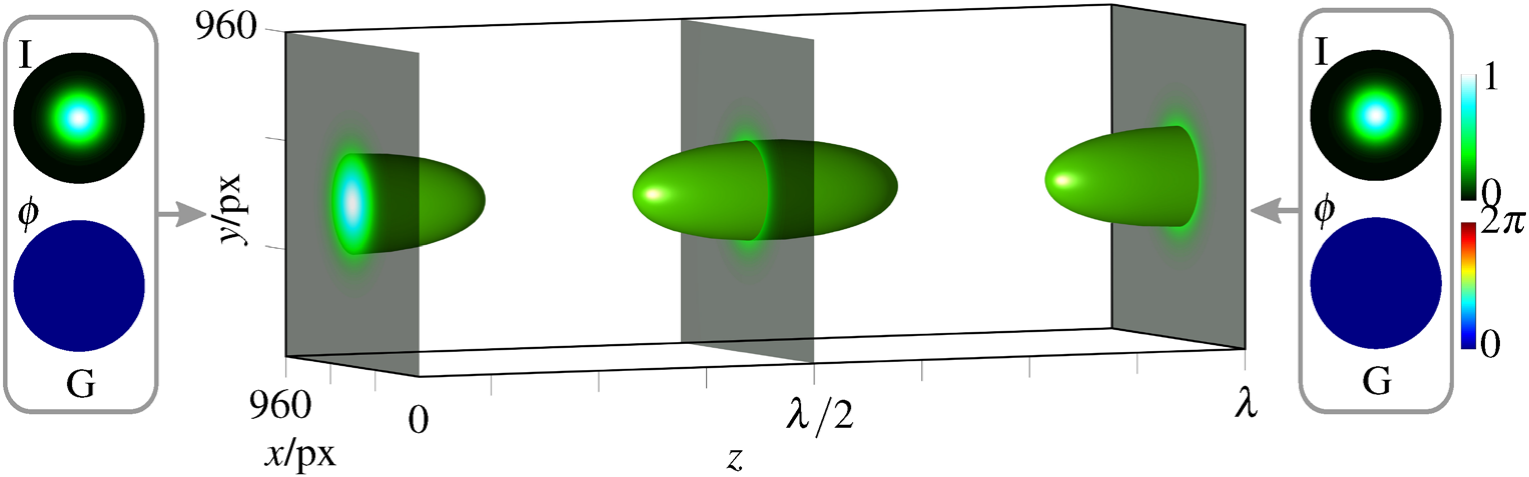
Numerical simulation of counter-propagating Gaussian beams (G). Left and right insets show intensity and phase, the 3d plot in the center shows a surface plot of 30% intensity of the counter-propagating superposition. Simulations are performed with MATLAB (MathWorks, R2018a), graphical design with Inkscape 0.92.

In order to shape light in 3d space, the use of single beams is limited to certain special cases where either fine structural changes only occur within the tight focus of the respective light field or spatial structures in the longitudinal direction are shaped far bigger than the wavelength^10,16,18,32^, as observed for, e.g., the optical conveyor beam based on Bessel mode modulation^25^. In contrast, the superposition of two separated counter-propagating beams yield a superior variety of light structures. It holds the potential of a strong longitudinal relative variation of phase as well as polarization of these beams within a propagation distance of one wavelength^29–31^, and an optional independent control of both beams. For this work, we exemplary choose to superimpose helical LG beams as well as CVBs, which are constituted of two (co-propagating) orthogonally polarized LG beams of opposite helical wave front^10,33^. We choose defined modes, to prevent arbitrary mixing that would lead to speckle patterns. Further description of these beam classes is given in the “Methods”. Based on the mode indices *l* and *n* of helical (h) LG modes ($\text {LG}^{\text {h}}_{n,l}$), the transverse amplitude and phase structure of the beam can be customized which in turn can be used to customize the transverse polarization pattern of CVBs. Due to the self-similarity, both beam classes do not change their transverse structure upon propagation, apart from scaling in aspect ratio due to divergence, which is beneficial for longitudinal extended light field generation. The 3d structured light field, realized by counter-propagation, can be described as

1 $$\begin{aligned} \vec {E}_{\text {cp}}= \vec {E_1}\cdot \exp \left( -\text {i}k_z z\right) + \vec {E}_2 \cdot \exp \left( \text {i}k_z z\right) \end{aligned}$$

with longitudinal wave number $k_z$ (wave vector $\vec {k} = [k_x,\,k_y,\,k_z]^T$). The superimposed self-similar light fields are represented by $\vec {E}_{1,2}$, both being scalar LG beams (see Methods Eq. (4); $\vec {E}_j = \vec {e}_j\,\text {LG}^{\text {h},j}_{n,l}$, with $j=\{1,\,2\}$ and $\vec {e}_j = \vec {e}_H = [1,\,0]^T$ or $\vec {e}_V = [0,\,1]^T$), of the same or orthogonal linear homogeneous polarization, or both CVBs [see “Methods” Eq. (7); $\vec {E}_j = \vec {\text {CVB}}_j$]. The exponential factors consider the paraxial propagation in $+z$- and $-z$-direction.

**Figure 2:**
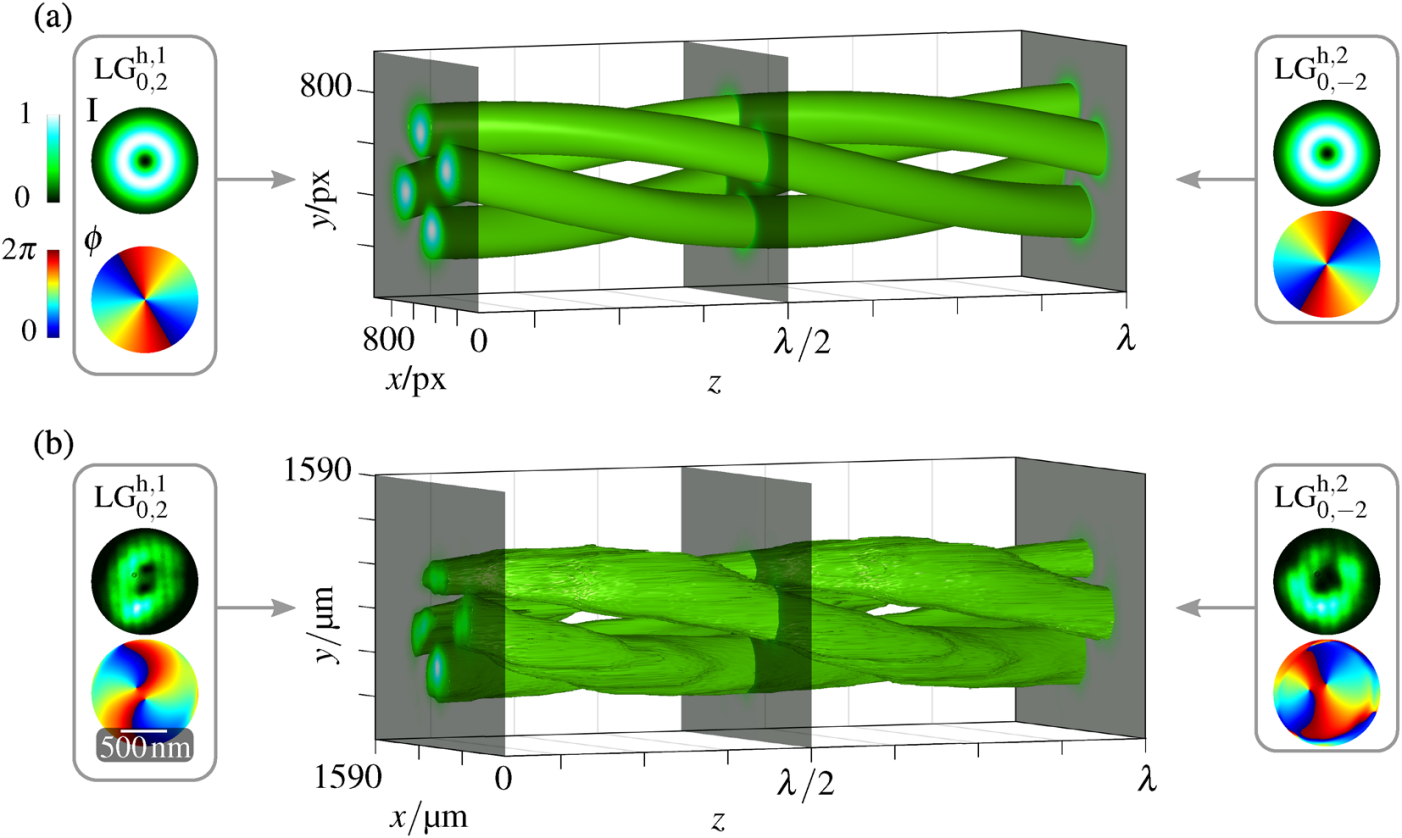
Numerical simulation and experimental measurement of the superposition volume of two counter-propagating LG beams. (**a**) shows numerically simulated intensity pattern within one wavelength ($\lambda =$532 nm) of the counter-propagating superposition of the beams depicted in the left and right inset. The colormaps for intensity (green) and phase (jet colors) are depicted in (**a**). The insets show the intensity pattern at the top and the phase pattern below. (**b**) shows the intensity pattern in experiment. The insets show the intensity of each beam at the top and the corresponding measured phase below. Simulations and analyses are performed with MATLAB (MathWorks, R2018a), graphical design with Inkscape 0.92.

It is well known, that counter-propagating fundamental Gaussian beams ($\text {G} = \text {LG}^\text {h}_{0,0}$) of the same polarization show longitudinal interference ($\vec {E}_j = \vec {e}_j \,\text {G}$, $\vec {e}_1 = \vec {e}_2$), which results in a sinusoidal longitudinal intensity variation, as illustrated in Fig. 1. Here and in the following, we denote intensity as $I = |\vec {E}_j|^2$ and global phase of each beam as $\phi$; 3d plots of intensity embed selected transverse planes and a 3d contour plot with surfaces at an intensity equal to 30% of the peak intensity created by MATLAB (MathWorks, R2018a; used for all simulations). In contrast, superimposing Gaussian beams with orthogonal polarization ($\vec {e}_1 \perp \vec {e}_2$) show no amplitude interference, i.e. the intensity stays constant, but “polarization interference”, causing a longitudinally varying polarization structure^34^. Both effects are due to longitudinal, global phase differences between counter-propagating beams. In our approach, we now additionally vary the transverse amplitude, phase and/or polarization structure of each beam $\vec {E}_{1,2}$ individually, adjustable by its mode indices. By this technique, relative amplitude, phase, and polarization differences of self-similar beams will be translated into a joint 3d structured light field of adaptable properties in the transverse as well as longitudinal spatial extent.

### LG beams of parallel polarization

First we consider the generation of a complex 3d light field based on superposition of two counter-propagating LG beams of the same polarization. Experimental details are given in the “Methods” section. We exemplarily analyze the superposition of two LG beams with a topological charge $l=\pm 2$ and without a radial node, i.e. $n=0$ , (LG$^{\text {h}}_{0,\pm 2}$). The transverse intensity *I* and phase $\phi$ pattern of each beam in its focal plane ($z=0$ in Eq. (4) in “Methods”) was determined numerically and measured experimentally, as depicted in the left and right boxes of Fig. 2a,b, respectively. The experimental beam profiles reveal a doughnut-shaped intensity and a vortex phase profile, carrying OAM, in good agreement with numerical simulations. There is no polarization modulation in this configurations, since both beams are linear horizontally polarized. The azimuthally varying phase structures reveal a topological charge of $l=\pm 2$, changing twice from 0 to $2\pi$, thus, (a) theoretically, the embedded central phase singularity is of higher-order ($l>1$). Higher-order phase singularities are naturally unstable upon minor perturbations^1^, causing its splitting into $|l|=2$ single-order phase singularities of $|l|=1$ in the (b) experimental realization. This also causes small deviations between numerically and experimentally determined intensity structure, however, the splitting effects mainly the central area of the beam which has low amplitude values. Thus, it will not disturb the generation of the expected 3d intensity structure. Slight asymmetries in the beams intensity structure, especially in $\text {LG}_{0,2}^{\text {h},1}$, are additionally caused by the beam splitter in the interferometric part of the setup. The pattern of experimental measured and numerically simulated 3d intensity distribution within the *z*-distance of $\lambda$ are shown in the middle part of Fig. 2a,b. With the same value of |*l*| and *n* for both beams, the Gouy phase^35,36^ of both beams is equal and, thus, the experimental counter-propagating light structure is extended into the centimeter regime, only limited by slight imperfections in the alignment. The experimental scanning procedure is described in the Methods section. 48 transverse measurements where performed within a *z*-distance of $\Delta z = \lambda$ and added to a continuous plot. The combination of a doughnut intensity shape and a vortex phase profile results in a spiral intensity structure based on transverse as well as longitudinal constructive and destructive interference. In longitudinal direction, the structure rotates by $360^\circ$ after a propagation distance of $2\lambda$.

#### Variation of spiral structures by mode indices

**Figure 3:**
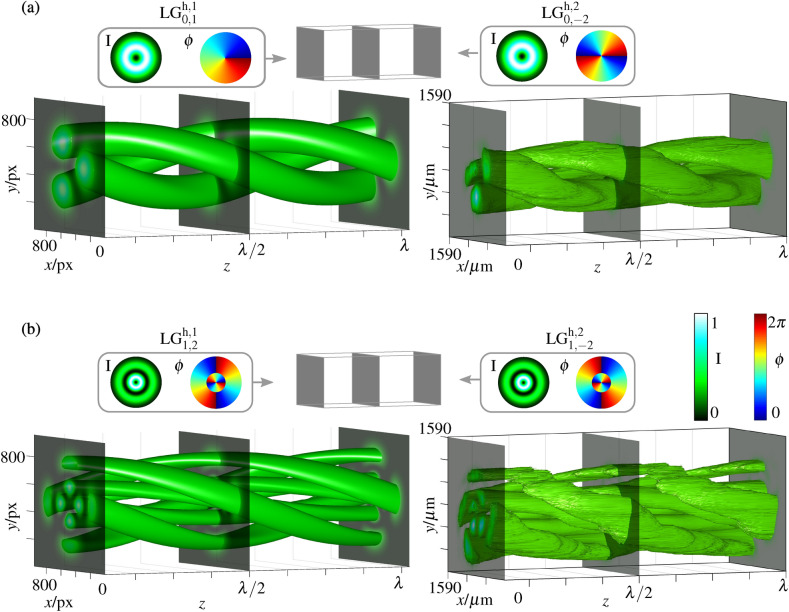
Simulation and experiment of index dependence for counter-propagating LG beams. Insets at the top on the left and right show intensity (left) and phase (right) profile of the respective input beam. LG$^{\text {h},1}_{n,l}$ and LG$^{\text {h},2}_{n,l}$ term the considered LG beams propagating in positive and negative *z*-direction, respectively, and their mode indices. Below, the 3d figure shows the intensity surface profile at 30% of peak intensity for the counter-propagating superposition volume within one wavelength (532 nm) in numerical simulation (left) and experiment (right). Subfigure (**a**) shows the variation of *l*, (**b**) depict a different radial mode number *n*. Simulations are performed with MATLAB (MathWorks, R2018a), graphical design with Inkscape 0.92.

Depending on the mode indices of combined LG modes, namely, the topological charge $l_{1,2}$ and radial mode number $n_{1,2}$, the realized spiralling structure can be adapted on demand. While *l* controls the azimuthal number of intensity maxima, *n* can be used to additionally shape radial amplitude nodes of each LG beam and, thereby, create additional rings. In total, the number of generated intensity maxima is given by

2 $$\begin{aligned} j_I=(n+1)\cdot l_\text {ges} \end{aligned}$$

with $l_\text {ges}=|l_1|+|l_2|$. Also for the customization of the spinning of the intensity structures upon propagation, the sum of the topological charges $l_1$ and $l_2$ is essential. It determines the number of twists of the intensity maxima within one wavelength given by

3 $$\begin{aligned} \varphi _I=\frac{2}{l_\text {ges}} \cdot 2\pi . \end{aligned}$$

The adaptability of the spiraling 3d intensity structures is illustrated by numerically simulated and experimental measured examples in Fig. 3. The given examples show that, with variation of *l*, the number of intensity spots and their rotation angle within one wavelength varies. In (a) an example of $|l_1| \ne |l_2|$ is given. Note that, in this case, the Gouy phase of counter-propagating beams is different, hence, the longitudinal extent of the spiraling intensity structure is limited. The effect of the second degree of freedom, *n*, is shown in the subfigure (b), for which $n\ne 0$ cause an additional ring on which intensity maxima are located. In total, for realized 3d intensity structures, the number of intensity maxima as well as the number of twists follow the rules in Eqs. (2) and (3).

#### Orthogonally polarized counter-propagating beams

**Figure 4:**
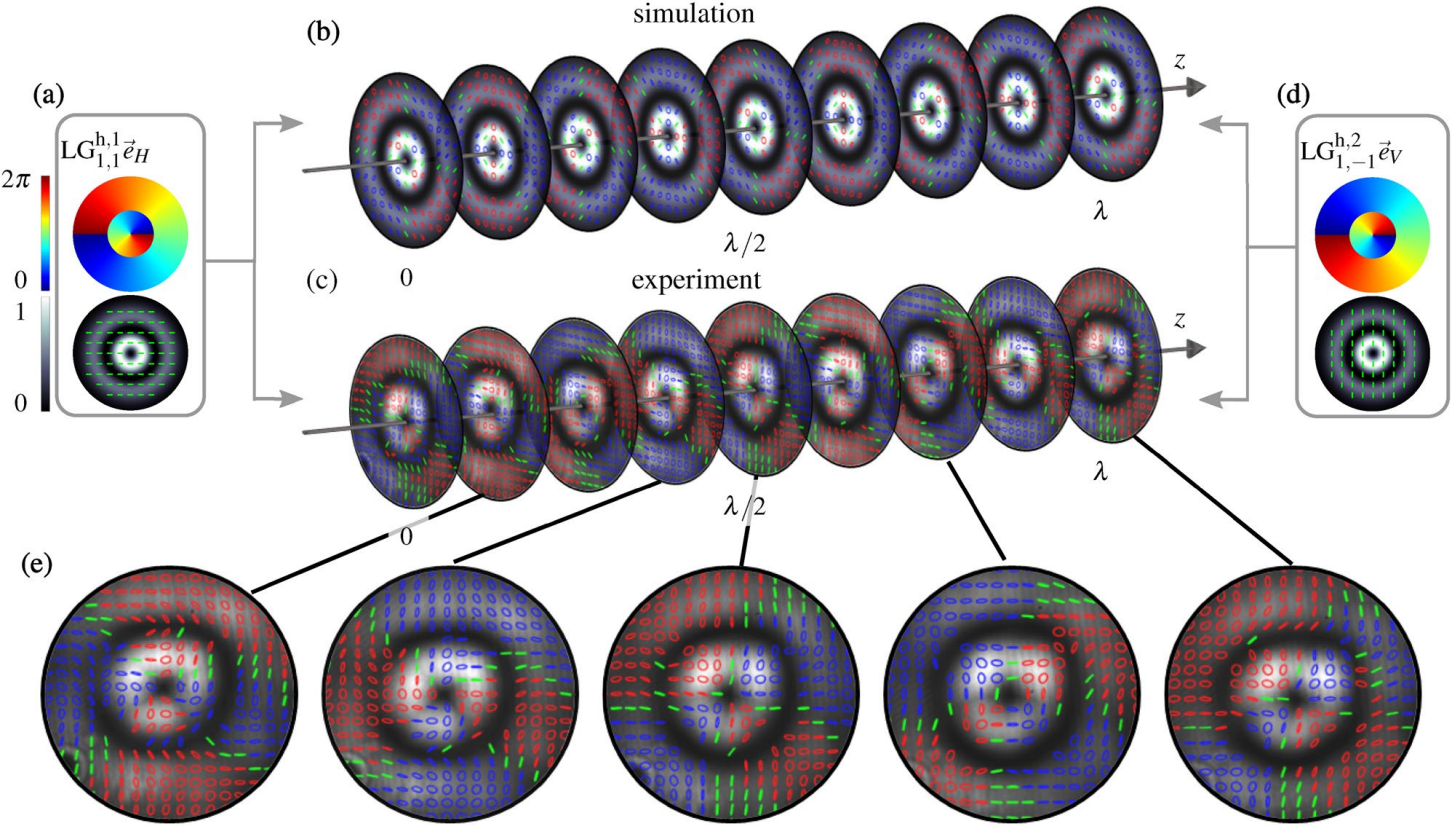
Numerical simulation and experimental measurement of the superposition volume of two counter-propagating orthogonally polarized LG beams. (**a**) and (**d**) show the numerical simulation of the beams coming from the left (**a**) and right (**d**) side. LG$^{\text {h},1}_{n,l}$ and LG$^{\text {h},2}_{n,l}$ term the considered LG beams propagating in positive and negative *z*-direction, respectively, and their mode indices. In the insets, at the top the transverse phase is depicted and below the intensity structure (background) with depicted polarization states (green). In (**b**) and (**c**), nine simulated and measured transverse planes of the polarization structure are shown, chosen equally distant within one wavelength *z*-distance ($\lambda =$532 nm) to visualize the behavior upon propagation. (**e**) Experimentally measured polarization distributions in representative transverse planes. The intensity structure is shown in grayscale with polarization ellipses on top (blue/red: left-/right-handed elliptical states; green: linear states). Simulations and analyses are performed with MATLAB (MathWorks, R2018a), graphical design with Inkscape 0.92.

In the previous section, spiraling intensity structures where realized on the basis of scalar, thus, amplitude and phase structured light fields of the same polarization. Thus, the realized 3d structured field was like-wise scalar. In the following, we aim for 3d shaping of a less investigated degree of freedom: the polarization of light. For this purpose, the counter-propagating beams will be of orthogonal polarization.

The exemplary set of beams consists of two orthogonally (linear horizontal and linear vertical) polarized LG beams of topological charge $l_1= -l_2 = 1$ and a radial node $n=1$. The intensity, phase, and polarization pattern of the input beams is depicted in the insets in Fig. 4a,d (simulations; top: $\phi$, bottom: *I* in grayscale with green polarization states). For each beam, the transverse phase pattern shows a vortex structure of charge $l = \pm 1$ including a phase jump of $\pi$ at the radius of the amplitude/intensity node. Hence, the intensity shows two centered rings while the polarization is homogeneous over the transverse plane. The light field, shaped by counter-propagation, is constant in its intensity profile but shows a modulation in polarization, as visible in Fig. 4b,c. Intensity and polarization in nine equally distributed transverse planes are shown within a *z*-range of a wavelength. Fig. 4 (**e**) visualizes the experimental measured polarization distribution of five representative transverse planes. The intensity profile at each position is shown in a grayscale colormap in the background, while polarization states are depicted by polarization ellipses with red/ blue and green highlighting right-/left-handed elliptical and linear states, respectively. In each plane an ellipse field with azimuthal change in ellipticity is found, varying from linear, via elliptical, to circular states and back (for left- and right-handed states each). This transverse evolution repeats twice in a full $360^\circ$ cycle around the optical axis. Upon propagation, this transverse pattern rotates and repeats itself after $\lambda /2$.

### Counter-propagating vectorial light fields

**Figure 5:**
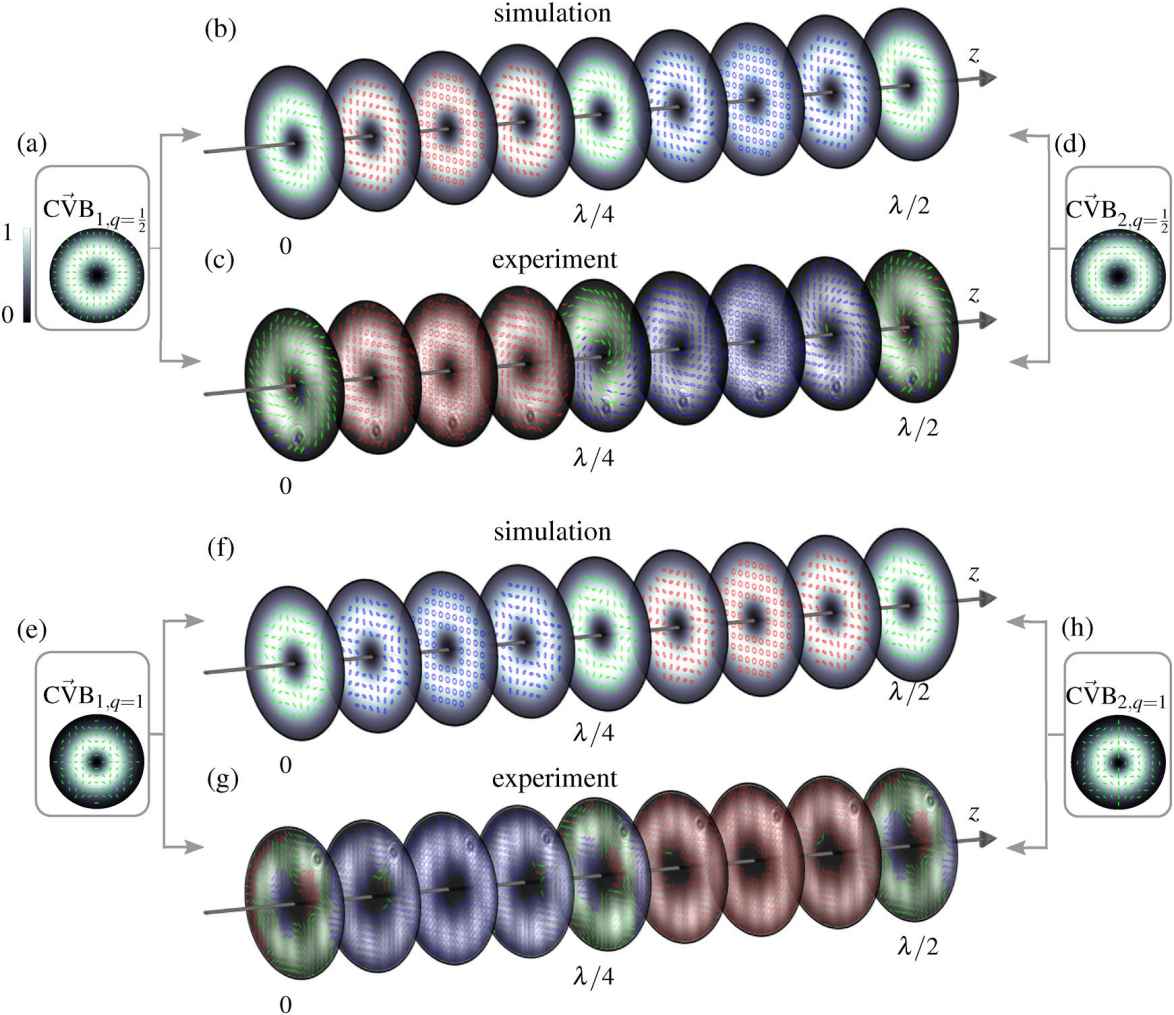
Numerical simulation and experimental measurement of the superposition volume of two orthogonal counter-propagating CVBs—(**a**)–(**d**) first-order CVBs ($q=1/2$), (**e**)–(**h**) higher-order CVBs ($q=1$). Insets on the left or right show the simulation of the input beams propagating in (**a**,**e**) positive ($\vec {\text {CVB}}_1$) or (**d**,**h**) negative ($\vec {\text {CVB}}_2$) *z*-direction. The intensity pattern is shown in grayscale in the background and the states of polarization are illustrated on top (green lines: linear states). Central images show (**b**,**f**) numerically simulated and (**c**,**g**) measured polarization structure within one wavelength (532 nm) propagation distance. Nine equidistant planes are shown with their intensity structure in grayscale and their polarization ellipses depicted at their respective transverse position (blue/red: left-/right-handed elliptical states). Simulations and analyses are performed with MATLAB (MathWorks, R2018a), graphical design with Inkscape 0.92.

Superimposing scalar LG beams, 3d changes realized in intensity or polarization originate from transverse and longitudinal differences in phase and intensity of LG beams, while polarization of each beam was kept constant. In the next step, we now additionally consider the transverse polarization structure, thus, transverse and longitudinal polarization differences of combined beams, as degree of freedom. For this purpose, we counter-propagate two vectorial light fields, more precisely, CVBs (Eq. (7) in “Methods”) and illustrate the enabled customization of 3d light fields by the example of orthogonally polarized radial and azimuthal ($\alpha = 0$ and $\pi /2$, respectively; $n=0$, $l=1$) as well as higher-order CVBs ($\alpha = 0$ and $\pi /2$; $n=0$, $l = -2$). The implemented experimental system is described in the “Methods” section below.

#### Counter-propagating first-order CVBs

Figure 5a–d shows numerical simulation and experimental measurement of the counter-propagating volume for first-order orthogonal CVBs ($q=1/2$, see “Methods”). In (a) and (d) the intensity and polarization structure of the input beams with (a) positive and (d) negative propagation direction are shown (simulation). The intensity profile (grayscale color in the background) forms a doughnut shape with radially or azimuthally pointing linear states of polarization (green) depicted in the foreground. Additionally, (b) the numerical simulation and (c) experimental measurement of the intensity and the polarization structure in the counter-propagating superposition is presented within a *z*-distance of a wavelength. Nine equally spaced transverse planes are depicted, showing the corresponding transverse intensity and polarization structure. Since we superimpose orthogonally polarized (radial and azimuthal) CVBs, no intensity interference occurs, thus the transverse intensity pattern stays constant, keeping its ring shape upon propagation. The polarization, however, changes significantly due to polarization interference, as it has also been shown for first-order CVBs in Ref. ^37^. In this case, we observe a 3d variation in polarization, going beyond the twisting of a transverse polarization pattern: While showing a CVB with transverse linear states spiraling around the beam center in one plane, it changes to become a scalar beam of pure circular polarization within the propagation distance of $\lambda /8$. In intermediate planes, elliptical states of polarization of varying orientation are found. This oscillation between vectorial and scalar light field is connected to paraxial spin-orbit interactions^37,38^.

#### Increasing the order of CVBs

Dependent on the chosen mode indices of counter-propagating CVBs, the resulting structure can easily be adapted, similar to the presented diversity of scalar LG beam superpositions. This is exemplified by increasing the order of counter-propagating CVBs, namely, we change to $l=-2$, keeping $n=0$ ($q=1$, see Methods). Results are presented in Fig. 5e–h. The counter-propagating higher-order CVBs of spider-web-like polarization structure are illustrated in (e) and (h) [$\alpha = 0$ and $\pi /2$, respectively; see Methods, Eq. (7)]. Similar to the previous example, the intensity stays in ring shape. The polarization, again, performs significant changes upon propagation: it varies from vectorial spider web structure to a scalar beam of circular polarization (after $\lambda /8$) and back to a vectorial spider web structure, which is rotated by 30°  at $\lambda /4$ propagation distance, thus, orthogonal to the distribution at $z=0$ or $\lambda /2$. Within the next $\lambda /4$ propagation distance, the polarization becomes the other handedness and ends again in a spider web of further 30°  rotation. Note that also for counter-propagating CVBs a further variation in mode indices is possible, which allows for an extension in number and structure of 3d shaped fields, while its longitudinal extent is again given by the differences in the Gouy phase of underlying LG beams.

## Conclusion and discussion

We take the customization of counter-propagating light fields to the next level, introducing an innovative approach to create and investigate 3d structured light fields generated by the counter-propagation of two self-similar scalar or vectorial beams. To overcome the experimental issue to measure counter-propagating light fields, we employ an artificial, digital counter-propagation of physically co-propagating light fields. This allows, for the first time to our knowledge, a detailed analysis of the counter-propagating superposition without any obstruction. By using Laguerre-Gaussian as well as orthogonal cylindrical vector beams, we are able to tailor the light in intensity and/or polarization in an extended 3d volume with longitudinal sub-wavelength precision and being at the same time fully adaptive to changing mode indices. The modulation in *x*- and *y*-direction is only limited by the resolution of the specific spatial light modulator employed, and its detection by the pixel size of the camera used, and not by the method itself. The *z*-dimension of the presented structures is only limited by the beam properties, namely by the Rayleigh distance and respective Gouy phase differences of combined self-similar beams, dependent on chosen mode indices. Experimentally, the high *z*-resolution is achieved by implementing an innovative digital, artificial counter-propagation, with its *z*-step size only being limited by the precision of the phase modulation of the SLM in the experimental system.

The 3d fields we exemplary present in this work are of special interest for applications that need longitudinally extended light structures, e.g. for advanced optical assembly of dielectric or polarization-sensitive particles in volumetric scenarios, or in atomic trapping taking advantage of 3d extended high-contrast intensity patterns. Furthermore, volumetric polarization modulation may give new insights into the fundamentals of singular light, for instance, the propagation dynamics of optical singularities^39,40^ in counter-propagating fields, classical entanglement^37^, or spin-orbit coupling^38^.

## Methods

### Laguerre-Gaussian beams and cylindrical vector beams

Helical Laguerre-Gaussian (LG$^\text {h}_{n,l}$) beams of radial mode number $n\in \mathbb {N}_0$ and azimuthal mode number (topological charge) $l \in \mathbb {Z}$ are represented by the complex function in polar coordinates $(r,\,\varphi ,\,z)$^36,41^

4 $$\begin{aligned} \text {LG}^{\text {h}}_{n,l}(r,\,\varphi ,\,z)&= A_{n,l}(r,z) \cdot \text {e}^{\text {i} \frac{k r^2}{2R(z)}}\cdot \text {e}^{\text {i}\phi _{n,l}^{G}(z)}\cdot \text {e}^{\text {i}l \varphi }, \quad \text {with} \end{aligned}$$

5 $$\begin{aligned} A_{n,l}(r,z)&= \sqrt{\frac{2n!}{\pi (|l|+n)!}}\cdot \frac{1}{w(z)}\cdot \text {e}^{-\frac{r{^2}}{w^2(z)}} \cdot \left( \frac{r\sqrt{2}}{w(z)}\right) ^{|l|}\cdot \text {L}_n^{|l|}\left( \frac{2r^2}{w^2(z)}\right) , \end{aligned}$$

6 $$\begin{aligned} \phi _{n,l}^G(z)&= (2n+|l|+1)\phi _{0,0}^G(z) \end{aligned}$$

(*k*: wave number, *R*(*z*): wave front curvature, *w*(*z*): beam radius, $w_0=w(0)$: beam waist). Here, $L_n^l(\cdot )$ represents the eponymous Laguerre polynomial^42^ and $\phi _{n,l}^G$ the Gouy phase shift of LG modes ($\phi _{0,0}^G$: Gouy phase of fundamental Gaussian beam). The factor $\exp (\text {i}l\varphi )$ gives the embedded phase vortex structure with on-axis phase singularity, both characterized by the topological charge *l*. As combination of helical LG beams, we consider cylindrical vector beams CVBs^10,33^ as

7 $$\begin{aligned} \vec {\text {CVB}} = \frac{1}{\sqrt{2}}\left[ \vec {e}_R\cdot \text {LG}^{\text {h}}_{n,l}\cdot \text {e}^{\text {i}\alpha }+ \vec {e}_L\cdot \text {LG}^{\text {h}}_{n,-l}\cdot \text {e}^{-\text {i}\alpha }\right] . \end{aligned}$$

Unit vectors $\vec {e}_{R,L}$ represent orthogonal right- and left-handed circular polarization states (Jones vectors) and $\alpha$ the phase relation between LG beams. Dependent on the choice of LG mode indices and phase relation $\alpha$, different transverse polarization structures are realized. For first-order CVBs, $|l|=1$ applies, whereas higher-order CVBs are realized for $|l|>1$.

### Digital counter-propagation

**Figure 6:**
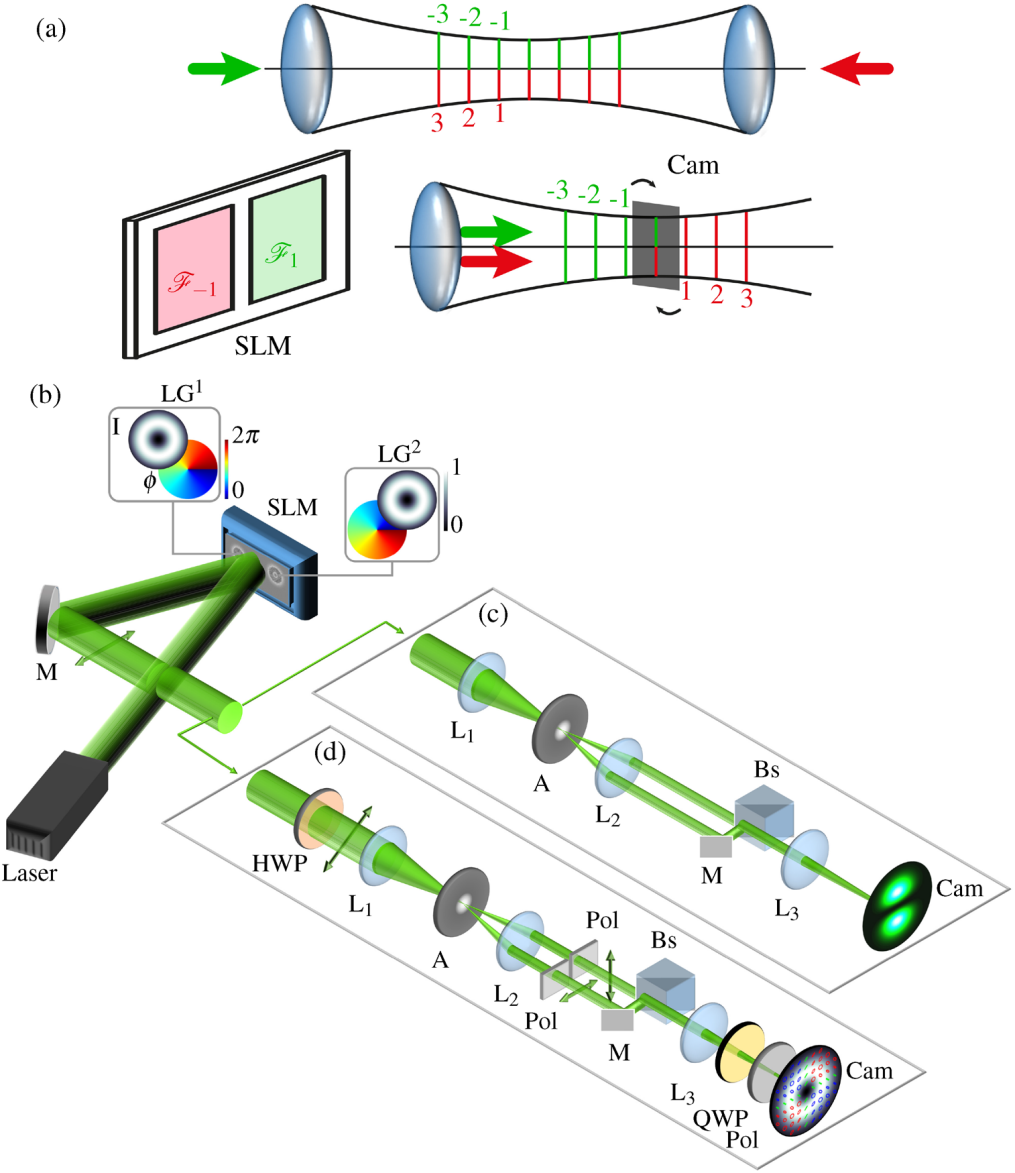
(**a**) compares the principle of counter-propagation at top with the digital counter-propagation method below. For digital counter-propagation, the propagation axes of both beams are superimposed, with beams propagating in the same *z*-direction, while they are shifted in or against propagation direction by adding the corresponding phase masks in the Fourier space on the SLM. Thereby, the typical counter-propagating interference patterns are generated in the plane of observation (Cam), as exemplarily shown for planes -1 and 1. (**b**) Experimental setup for the investigation of counter-propagating scalar LG beams. Amplitude and phase modulation via a phase-only SLM (Holoeye Pluto) to generate two LG beams by two side-by-side Fourier holograms. (**c**) Co-propagating superposition and intensity analysis of modulated beams of the same horizontal linear polarization. (**d**) Orthogonally polarizing modulated beams by half wave plate (HWP) and polarizers (Pol) and subsequent co-propagating superposition. Superposition is analyzed via Stokes polarimetry by rotatable quarter wave plate (QWP) and fixed polarizer (Pol) in front of the camera (Cam, Ueye SE (UI-1240SE)). Laser: Nd:YAG, 532 nm; M: mirror, L: lens (focal distances $\{{f}_1,\,{f}_2,\,{f}_3\}=\{200,\,100,\,200\}\,\text{ mm }$), A: aperture, Bs: beam splitter. 3d visualization created using PowerPoint Professional Plus 2019 v. 1808, graphical design with Inkscape 0.92.

For the experimental analysis of counter-propagating light fields, a special approach is required. In a spatial counter-propagating superposition the light field cannot be observed on the beam axis, as the required detector, e.g. a camera, would prevent the interaction of the two counter-propagating light fields by spatially blocking one beam. Thus, the 3d structured volume would not form on the camera detector. However, there exist mathematical ways to enable a transverse measurement of the counter-propagating light field. Applying this, we implemented a digital, artificial counter-propagation approach based on a spatially co-propagating superposition^37^. Digital propagation is based on the angular spectrum representation of light according to which a scalar propagated light field in real space can be described as $U(\vec {r},z)=\mathcal {F}^{-1}\{\mathcal {F}[U(\vec {r},0)]\cdot \exp (\text {i}k_z z)\}$, with $\mathcal {F}^{-1}$ representing the (inverse) Fourier transform, $\vec {r}=(x,y)$ are the transverse coordinates in real space^43^. Hence, by implementing a Fourier hologram of the scalar field at $z=0$ ($\mathcal {F}[U(\vec {r},0)]$) with additional propagating phase factor $\exp (\text {i}k_z z)$, the light field in real space can easily be propagated digitally in $\pm z$-direction. This additional propagation phase factor is calculated based on the given formula including the desired *z* position and $k_z=\sqrt{k^2-k_x^2-k_y^2}$. Finally the result is added as a phase term to the SLM hologram.

To take advantage of this approach, we realize superimposed LG beams or CVBs individually by Fourier holograms^44^ on a phase-only spatial light modulator (SLM) or Fourier holograms in combination with a q-plate^45^, respectively. The realized two self-similar beams physically co-propagate and meet on the camera (Cam) to form the same interference pattern as the counter-propagating configuration at a specific *z*-position. By digital propagation of one of the beams in $+z$- and the other one in $-z$-direction, the whole 3d volume of the counter-propagating superposition can be scanned. The comparison of counter-propagation and its digital counterpart is visualized in Fig. 6a. While for the analysis of the intensity structure of the customized field the camera in the observation plane is sufficient, for polarization structures a Stokes polarimetry is performed, utilizing a quarter wave plate and a polarizer in front of the camera^46^.

### Experimental analysis of counter-propagating scalar light fields

The self-similar beams are generated by a single SLM (Holoeye Pluto) divided into two separated modulation regions that display Fourier holograms, as shown in Fig. 6b. The SLM is illuminated with a significantly expanded, collimated beam to ensure equivalent power of both realized beams. The phase-only holograms embed amplitude as well as phase information using the weighted blazed grating approach^47^. At this stage the digital propagation phase factor is added in the hologram, when the beams are propagated. After spatially filtering the first diffraction order by the lenses L$_1$ and L$_2$ in combination with an aperture (A), the two beams are superimposed on-axis in a co-propagating configuration by a beam splitter (Bs) and a mirror (M; see Fig. 6c). Lens L$_3$ focuses the superimposed beams to form the desired real-space superposition of artificially counter-propagated beams on the camera. The Fourier relation of SLM and observation plane is realized by an odd number of imaging lenses. The scanned volume has an extension of $(1.6\times 1.6)\,\text{ mm}^2$ in transverse extent and one wavelength (532 nm) along the propagation axis.

For experimental investigation of orthogonally polarized beams, the setup is adapted as shown in Fig. 6d. A half wave plate (HWP) rotates the originally horizontal linear state of polarization to $45^\circ$ for both beams. Two linear grid polarizers (Pol), horizontally and vertically oriented, set the state of polarization for each beam individually, such that they are of orthogonal linear polarization. A beam splitter (Bs) and a mirror (M) merges both beams on a joint propagation axis. The polarization analysis for the 3d structured light field is facilitated by a Stokes polarimetry system^46^ consisting of a rotatable quarter wave plate (QWP) and a horizontal polarizer (Pol) in front of the camera (Cam). To determine the Stokes vector for each camera pixel, we perform 12 measurements for different angles of the quarter wave plate in the range of $0^\circ$ to $180^\circ$. Repeating this measurement for all desired *z*-positions results in a 3d profile of the polarization.

### Experimental analysis of counter-propagating vectorial light fields

**Figure 7:**
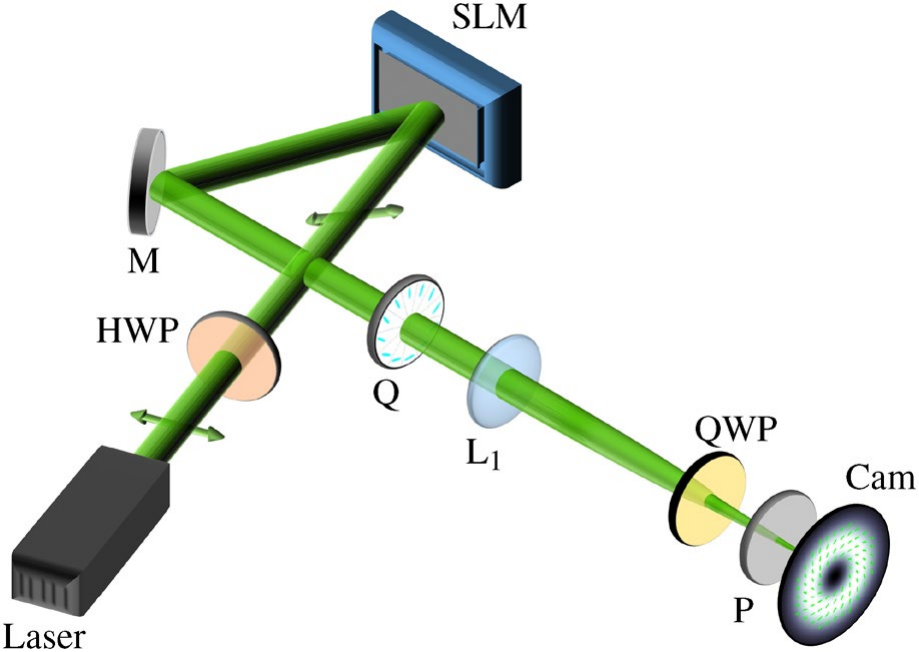
Experimental setup for creation of polarization-modulated digital counter-propagating light fields. A horizontally linear polarized Gaussian beam, adaptable by the phase-only SLM and a vertically linear Gaussian beam, unaffected by SLM modulation, are created, both propagating on the optical axis. The SLM is only responsible for digital counter-propagation, while the q-plate transforms Gaussian beams into two co-propagating CVBs. HWP: half wave plate, M: mirror, Q: q-plate (Thorlabs WPV10L-532 m=1), L: lens (f$_1=200$), QWP: rotatable quarter wave plate, P: polarizer, Cam: camera. 3d visualization created using PowerPoint Professional Plus 2019 v. 1808, graphical design with Inkscape 0.92.

To create cylindrical vector beams (CVBs) and analyze their counter-propagation experimentally, we apply a combination of SLM (Holoeye Pluto) and q-plate ($q=1/2$ or $q=1$)^45^ (see Fig. 7). The horizontal linear polarization of the initial Gaussian laser beam (expanded and collimated) is tilted to $45^\circ$ before it is incident on the SLM. In the SLM configuration at hand, only the horizontally polarized components can be shifted digitally in phase by the hologram while the vertically polarized part will stay unaffected. Thus, we can treat the horizontal and vertical polarization states as if they were as separate beams, co-propagating on-axis. Next, a q-plate is used, which converts the homogeneous polarization depending on its input state into a spatially varying polarization pattern—it approximately converts scalar fundamental Gaussian beams into CVBs. In the case at hand, for the q-plate of order $q=1/2$ (Thorlabs, WPV10L-532), the beam of horizontal polarization is transferred into a radially polarized CVB and one of vertical polarization into an azimuthally polarized CVB, and, for a q-plate of order $q=1$ (Thorlabs, WPV10-532), into orthogonal higher-order CVBs of spider-web-like polarization structure^48^. The lens ensures the Fourier relation between SLM and observation plane, required for digital propagation of CVBs. The resulting polarization pattern per *z*-slice of the counter-propagating CVBs is analyzed by the Stokes polarimetry.

Note that in the experimental configuration we can only digitally propagate the initially horizontally polarized beam, thus, one of the CVBs by the SLM. Hence, we shift this beam about twice the distance, while the other CVB stays at the same position. Due to the self-similarity of CVBs, this approach gives the same results as digitally counter-propagating both of the beams.

## Data Availability

Data underlying the results presented in this paper are not publicly available at this time but may be obtained from the authors upon reasonable request.
